# Neural Circuit Remodeling: Mechanistic Insights from Invertebrates

**DOI:** 10.3390/jdb12040027

**Published:** 2024-10-11

**Authors:** Samuel Liu, Kellianne D. Alexander, Michael M. Francis

**Affiliations:** 1 Department of Neurobiology, University of Massachusetts Chan Medical School, Worcester, MA 01605, USA; 2 Program in Neuroscience, University of Massachusetts Chan Medical School, Worcester, MA 01605, USA

**Keywords:** *C. elegans*, *Drosophila*, neural circuits, remodeling, synapse elimination, transcriptional regulation, cell adhesion, cellular activity

## Abstract

As nervous systems mature, neural circuit connections are reorganized to optimize the performance of specific functions in adults. This reorganization of connections is achieved through a remarkably conserved phase of developmental circuit remodeling that engages neuron-intrinsic and neuron-extrinsic molecular mechanisms to establish mature circuitry. Abnormalities in circuit remodeling and maturation are broadly linked with a variety of neurodevelopmental disorders, including autism spectrum disorders and schizophrenia. Here, we aim to provide an overview of recent advances in our understanding of the molecular processes that govern neural circuit remodeling and maturation. In particular, we focus on intriguing mechanistic insights gained from invertebrate systems, such as the nematode *Caenorhabditis elegans* and the fruit fly *Drosophila melanogaster*. We discuss how transcriptional control mechanisms, synaptic activity, and glial engulfment shape specific aspects of circuit remodeling in worms and flies. Finally, we highlight mechanistic parallels across invertebrate and mammalian systems, and prospects for further advances in each.

## 1. Introduction

Developing neurons often substantially restructure their connections in order to optimize circuit performance in the mature nervous system. This process is known as synaptic remodeling and occurs throughout the animal kingdom. Synaptic remodeling in the mammalian brain is thought to be largely achieved through a process known as pruning [1,2,3], though recent work suggests the importance of alternate mechanisms [4]. During pruning, specific synapses or synaptic debris are removed by neighboring glial cells through phagocytosis or related mechanisms like trogocytosis. In humans, synapse density peaks near birth and declines throughout adolescence as synapses are pruned, before stabilizing during the third decade of life [5]. Deficits in synaptic pruning and circuit remodeling in humans are linked with a variety of neurodevelopmental and neuropsychiatric disorders, including both autism spectrum disorders and schizophrenia [1,6]. Similar programs for synaptic remodeling sculpt the connectivity of developing neural circuits in mammalian models and invertebrates such as the nematode *Caenorhabditis elegans* [7,8,9,10] and the fruit fly, *Drosophila melanogaster* [11]. Studies of these invertebrate models have provided new and complementary information about the molecular programs that direct synaptic remodeling. Here we review recent advances in our mechanistic understanding of synaptic remodeling with a focus on insights from studies in invertebrate systems.

**Figure 1:**
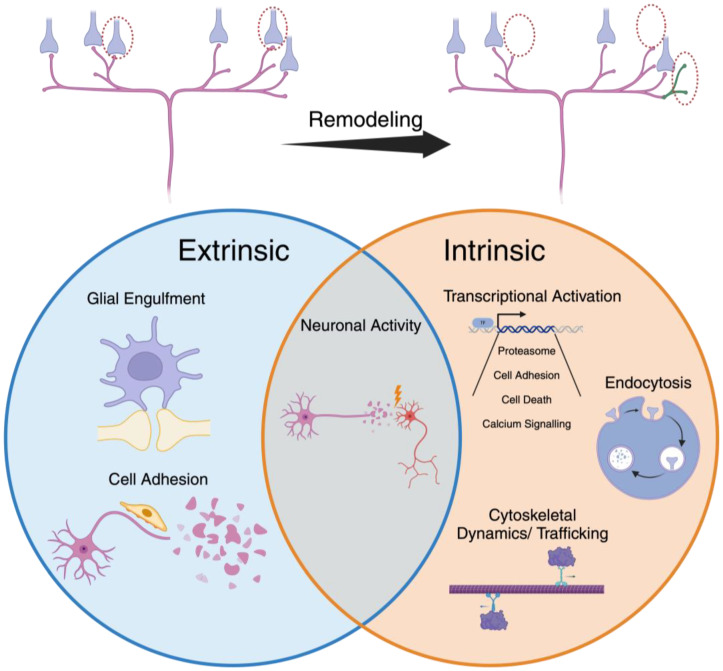
Both neuron-extrinsic and -intrinsic factors shape synaptic remodeling. During synaptic remodeling, juvenile synaptic connections are either eliminated or maintained to achieve adult-specific connectivity. Upper, synaptic connections are removed (red circles), maintained or expanded (green) during remodeling. Lower, neuron-intrinsic processes include endocytosis, cytoskeletal dynamics, and transcriptional activation of genes important for protein degradation through the ubiquitin–proteasome system or for cell adhesion. Extrinsic regulation occurs primarily through glial engulfment of synaptic material. Neuronal activity may be important for regulation of both intrinsic and extrinsic processes. See text for additional details. Created with BioRender.com (accessed 13 August 2024).

During remodeling, specific synapses are eliminated while others are stabilized or expanded, highlighting the importance of mechanisms for both synapse disassembly and assembly. Moreover, both neuron-intrinsic and -extrinsic factors can shape remodeling (Figure 1). For example, transcriptional regulation of neuron-intrinsic factors for synapse disassembly is important for the elimination of glutamatergic synapses in relay neurons of the mouse lateral geniculate nucleus [12,13,14]. In contrast, cell-extrinsic regulation through phagocytic engulfment by neighboring glia has been implicated in shaping the connectivity of circuits in the mouse visual cortex [15,16,17]. In many cases, however, the specific mechanisms that engage circuit remodeling and specify its timing remain unclear. Likewise, we are only beginning to understand how cell-intrinsic and -extrinsic processes may cooperate to orchestrate circuit remodeling. Invertebrate models such as *C. elegans* and *Drosophila* have proven to be excellent systems for studying the molecular and cellular processes driving developmental remodeling. In the case of *C. elegans*, the translucency of the cuticle greatly simplifies the fluorescent imaging of neurons and synapses in intact animals. In addition, the simple and largely invariant organization of the nervous system and well-characterized *C. elegans* developmental trajectory are major assets for studies of circuit connectivity. Similarly, the extensive rewiring during metamorphosis and powerful genetic tools available in *Drosophila* offer attractive experimental strengths for uncovering molecular programs underlying developmental rewiring.

## 2. Synapse Elimination and Reorganization during Developmental Rewiring of *C. elegans* Neural Circuits

**Figure 2:**
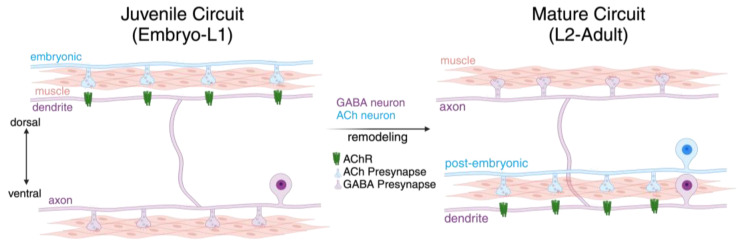
Remodeling of *C. elegans* DD GABAergic neurons. Schematic of motor circuit. Left, in the juvenile circuit, embryonic DD motor neuron dendrites (purple) receive input (green receptors) from embryonic-born cholinergic motor neurons (blue terminals) in the dorsal nerve cord. Juvenile DD motor neurons have GABAergic synaptic outputs (purple terminals) onto ventral body wall muscles (pink). Right, during the L1-L2 transition, DD motor neurons are remodeled to receive inputs from post-embryonic-born cholinergic motor neurons (blue) in the ventral cord. The DD motor neuron outputs (purple terminals) are relocated dorsally, forming new synaptic contacts onto the dorsal body wall muscle.

The mature *C. elegans* nervous system is produced through extensive developmental remodeling of juvenile neural circuits. *C. elegans* development proceeds through four larval stages prior to adulthood. Nearly a third of the *C. elegans* hermaphrodite nervous system (80 of 302 neurons) is formed post-embryonically and integrated into pre-existing juvenile neural circuits after the first larval stage. More than fifty of these post-embryonic-born neurons are motor neurons, necessitating the widespread reorganization of juvenile motor circuits to accommodate these neurons’ emergence [18,19]. The most striking example of this synaptic reorganization is the synaptic remodeling of GABAergic dorsally directed (DD) motor neurons [20,21,22,23], which swap both pre- and postsynaptic partners during a roughly 10 h period following the first stage of larval development [7,8,9,10] (Figure 2). Immediately after hatching, juvenile cholinergic synaptic inputs to GABAergic DD neurons are located dorsally, while juvenile DD synaptic outputs onto muscles are located ventrally. During remodeling, the dorsal synaptic inputs formed in the embryo are eliminated, and post-embryonic-born (VA/VB) cholinergic neurons establish new synaptic inputs to DD neurons ventrally. In parallel, juvenile ventral GABAergic DD presynaptic release sites are removed from ventral muscles and relocated to the dorsal side where new inhibitory outputs to muscles are formed [10,24]. Synaptic remodeling proceeds without obvious changes in the morphological features of DD neurons [10,18,23,25,26]. Neuron-extrinsic processes involved in large-scale neuron structural modifications in other systems, such as the engulfment of axonal material by neighboring glia [15], are therefore less critical in this system. Instead, the remodeling of DD neurons relies more strongly on neuron-intrinsic pathways. In the following sections, we detail the molecular mechanisms implicated in the developmental reorganization of GABAergic connectivity in the motor circuit, focusing on processes impacting the rearrangement of presynaptic or postsynaptic sites. 

## 3. Developmental Redistribution of Presynaptic GABA Release Sites

Efforts from numerous research groups have identified factors important for the relocation of GABAergic release sites from the ventral to dorsal processes of DD neurons during circuit remodeling [7]. This surprising phenomenon was first documented in groundbreaking electron microscopy reconstruction studies conducted over 40 years ago [10,27]. In these studies, the locations of synaptic contacts were primarily identified from the positioning of synaptic vesicle clusters in the DD neuronal processes. Follow-up studies have largely relied on fluorescent imaging of genetically encoded synaptic vesicle reporters expressed in GABAergic neurons of intact *C. elegans* to investigate presynaptic remodeling. For this approach, synaptic vesicle proteins such as RAB-3 or SNB-1/synaptobrevin are fused with fluorescent reporters such as GFP or mCherry and expressed specifically in DD neurons [28,29,30]. Many researchers have exploited fluorescent imaging of intact *C. elegans* in combination with the powerful genetic tools available in this system to reveal the key effectors of synaptic remodeling [10,18]. We summarize several key findings from these studies in the following sections.

### 3.1. Transcriptional Mechanisms Controlling Synaptic Remodeling

The heterochronic gene *lin-14* was the first to be implicated in the control of DD neuron remodeling. *lin-14* had previously been shown to be important for the timing of developmental events in other tissue types [31,32] and was later shown to encode a BEN domain transcriptional regulator [33,34]. Work from the Jin laboratory demonstrated that *lin-14* also regulates the timing of synaptic remodeling in DD neurons [35]. Specifically, SNB-1::GFP labeled synaptic vesicle assemblies relocated precociously in the DD neurons of *lin-14* loss-of-function mutants, indicating that LIN-14 acts to delay presynaptic remodeling. Another heterochronic gene, the hunchback-like transcription factor *hbl-1*, has also been implicated in controlling the timing of remodeling. Mutation of *hbl-1* delays GABAergic DD remodeling [36]. In contrast, increased neuronal activity or mutation of microRNA miR-84 induce increased *hbl-1* expression and precocious remodeling [36], suggesting that miR-84 normally acts as a negative regulator of *hbl-1* expression. Together, these studies suggest temporally controlled LIN-14 and HBL-1 activities work in opposition to govern the timing of presynaptic remodeling, where elevated *lin-14* expression inhibits remodeling while elevated *hbl-1* expression promotes the initiation of GABAergic DD remodeling.

The Pitx homeodomain transcription factor UNC-30 is a terminal selector required for GABAergic identity in *C. elegans* [37,38,39], but also controls the expression of key genes that can either promote or impede synaptic remodeling [40,41]. For example, UNC-30 promotes the expression of the Iroquois-like homeodomain transcription factor, IRX-1, which affects the timing of DD remodeling [42,43]. While the mutation of *unc-30* impacts several aspects of remodeling and synaptic patterning [44], the knockdown of *irx-1* delays remodeling [42,43]. Additional pro-remodeling transcription factors include the myelin gene regulatory factor family proteins (MYRF). MYRF-1 and MYRF-2 are cleaved from the ER membrane into active N-terminal fragments that translocate into the nucleus where they act redundantly to control the reorganization of GABAergic presynaptic release sites [45]. While *myrf-1;myrf-2* double mutants show delayed remodeling, overexpression of active N-terminal MYRF fragments produce accelerated remodeling [45]. The leucine-rich repeat transmembrane protein PAN-1 is required for the stabilization of MYRF at the cell membrane and the subsequent cleavage of the N-terminal fragment that is translocated to the nucleus [46]. Notably, a recent study provides intriguing evidence that MYRF-1 is necessary for expression of the microRNA *lin-4*, which controls developmental timing through post-transcriptional downregulation of LIN-14 [47].

In contrast to the TFs discussed above, the COUP-TF nuclear hormone receptor UNC-55 is not expressed in DD neurons. Instead, *unc-55* is highly expressed in a post-embryonic-born population of GABAergic motor neurons, the ventrally directed (VD) GABAergic neurons, that do not undergo remodeling [48,49]. Interestingly, mutation of *unc-55* produces ectopic remodeling of the VD neurons, implicating UNC-55 as a repressor of remodeling. As GABAergic VDs begin to form, *unc-55* expression increases and subsequently suppresses the expression of transcription factors that promote remodeling, such as *irx-1* and *hbl-1* [36,41,43]. In *unc-55* mutants, VD neurons form ectopic synapses with dorsal muscles [36], and ectopic expression of *unc-55* in larval stage 1 (L1) DD GABAergic neurons is sufficient to inhibit the remodeling of their presynaptic release sites [40]. Recent chromatin immunoprecipitation sequencing (ChIP-seq) studies identified a group of roughly 1300 genes that are co-regulated by UNC-30 and UNC-55, pointing toward key biological pathways regulated during remodeling (discussed in more detail below) [41]. For instance, UNC-55 suppresses the expression of targets *irx-1* [42] and *unc-8* [43], and enhances the expression of *pde-4* [41]. The identification and characterization of transcription factors important for remodeling has clarified the transcriptional network that determines the timing of remodeling, but an overarching model that integrates these various transcriptional pathways and their downstream mechanisms has yet to fully emerge.

### 3.2. Cellular Mechanisms of Synaptic Remodeling

Genetic disruption of exocytosis has implicated neurotransmitter signaling in regulating the timing of presynaptic remodeling [36]. In addition, optogenetic activation of L1 GABAergic DD neurons was shown to accelerate presynaptic DD remodeling [50]. At least some of this activity-dependence occurs through activity-dependent regulation of the HBL-1 transcription factor described above [36]. However, additional evidence for the importance of neuronal activity comes from recent studies of the epithelial sodium channel ortholog channel, UNC-8/DEG/ENaC. *unc-8* was initially identified as an effector of remodeling from an elegant RNAi-based screen to identify genes required for the ectopic remodeling of VD neurons in *unc-55* mutants [43]. Genetic studies provided evidence that calcium influx mediated through UNC-8 in combination with the P/Q-type voltage-gated calcium channel UNC-2 activates the calcium/calmodulin phosphatase Calcineurin (CaN) [50]. Mutations that impair CaN function delay presynaptic removal [50]. Further analysis suggested that UNC-8-mediated removal of presynaptic sites is partially dependent on the apoptotic cell death adaptor protein CED-4/Apaf1 [50,51]. Notably, findings from a prior study had also identified components of the apoptotic cell death pathway from a forward genetic screen to identify defects in the remodeling of presynaptic sites [51]. These studies showed that synaptic vesicle removal is delayed by the mutation of the *ced-3*/Caspase-3 gene. The isolation and characterization of a phenotypically similar mutant implicated the actin-filament-severing protein, GSNL-1/Gelsolin, downstream of CED-3. Further analysis suggested a model where GSNL-1 cleavage by CED-3 is required for presynaptic F-actin disassembly during the removal of GABAergic presynaptic release sites. When considered together with the findings for UNC-8 discussed above, it is tempting to speculate that UNC-8-dependent calcium influx may contribute toward the initiation of this process.

The importance of UNC-8 in presynaptic remodeling was further reinforced by additional studies linking UNC-8 with parallel activation of activity-dependent bulk endocytosis (ADBE) through the actions of CaN. ADBE is a clathrin-independent mechanism for membrane recycling that involves CaN dephosphorylation of several targets including the DYN-1/dynamin GTPase [52,53,54,55,56,57]. Several components of the ADBE pathway, including DYN-1, the F-BAR protein SNDP-1/syndapin, and components of the Arp2/3 complex involved in the polymerization of branched actin, were also shown to be important for the UNC-8-dependent removal of GABAergic DD presynaptic components and the subsequent generation of new presynaptic release sites [52]. Interestingly, *unc-8* expression is dependent on *irx-1*, offering a potential link between the mechanisms for transcriptional activation and cellular effectors of presynaptic disassembly [22].

As noted above, additional potential links between transcriptional regulation and cellular effectors of remodeling emerged from ChIP-seq studies that identified more than 1300 putative shared targets of the UNC-55 and UNC-30 transcription factors [41]. In particular, genes important for cyclic AMP (cAMP) metabolic processes were enriched as common targets between the two transcriptional regulators of remodeling. For instance, the phosphodiesterase *pde-4*/PDE4B was amongst the genes with the strongest UNC-30 and UNC-55 binding signals. Additional characterization showed that *pde-4* mutants exhibit premature remodeling, implicating the regulation of intracellular cAMP levels as another key signal in setting the timing of remodeling.

In addition to mechanisms that establish the timing of remodeling or processes involved in presynaptic disassembly, several studies have revealed key features required for the relocation of presynaptic release sites during remodeling. The onset of remodeling is correlated with an increase in microtubule dynamics without obvious changes in microtubule polarity [58]. The stabilization of microtubules (MT) via genetic manipulation [58] or accumulation of intermediate filaments (IF) [59] prevents presynaptic remodeling, reinforcing the importance of cytoskeletal dynamics in remodeling events [58,59,60,61]. The importance of MT dynamics during remodeling is further underscored by the finding that disassembled synaptic material is trafficked along microtubules during remodeling and reused in the construction of new presynaptic release sites [62]. The transport of presynaptic material to newly established release sites is mediated by the Kinesin-3 plus end motor UNC-104 and facilitated by cyclin-dependent kinase CDK-5 [62].

These and additional studies have elucidated key genetic pathways underlying presynaptic remodeling, dramatically expanding our understanding of the neuron-intrinsic mechanisms that drive this process (Table 1). An important next step will be to determine how these processes may be interconnected and orchestrated at the cellular level to achieve the rapid presynaptic remodeling that is observed in GABAergic DD neurons. In comparison, our understanding of molecular events central to the remodeling of postsynaptic sites on DD neurons is less well developed, though an intriguing picture has begun to emerge from recent studies.

### 3.3. Removal and Redistribution of Postsynaptic Sites on GABAergic DD Neurons

GABAergic DD neurons receive synaptic inputs primarily from cholinergic motor neurons. During remodeling, juvenile cholinergic synaptic inputs located on the dorsal DD neurites are eliminated (Figure 2). Simultaneously, new inputs from post-embryonic-born cholinergic motor neurons are formed on ventral neurites to establish mature connectivity. The locations of postsynaptic sites associated with synaptic inputs to DD neurons have been defined using cell-specific expression of GFP-tagged acetylcholine receptor (AChR) subunits [20,23,26,42,44,63]. Interestingly, studies to date suggest that the molecular events controlling the remodeling of these synaptic inputs are largely distinct from those previously defined in the rewiring of GABAergic outputs. For example, the genetic disruption of synaptic vesicle release or caspase function have each been shown to delay presynaptic remodeling, but do not significantly alter postsynaptic remodeling [20,50,51].

Efforts to gain a mechanistic understanding of postsynaptic remodeling have largely focused on the removal of juvenile postsynaptic sites in GABAergic DD dendrites. In particular, a recent study implicated the homeodomain transcriptional regulator DVE-1 from a forward genetic screen for mutants in which the elimination of juvenile dorsal postsynaptic sites is impaired [20]. In wild-type animals, postsynaptic receptor clusters are located exclusively to the dorsal side in the juvenile circuit but are completely removed during remodeling. As remodeling progresses, new postsynaptic receptor clusters are established ventrally, indicating a transition to mature circuit connectivity. In *dve-1* mutants or with temporally controlled degradation of neuronal DVE-1 protein, the removal of dorsal juvenile postsynaptic sites is impeded. With impaired DVE-1 function, these synapses remain structurally and functionally intact well into adulthood [20]. Cell-autonomous expression of *dve-1* in GABA neurons was shown to be sufficient for synapse elimination to proceed, suggesting a model where DVE-1 transcriptional regulation in GABA neurons is required for synapse elimination [20]. DVE-1 ChIP-seq analysis showed that components of the ubiquitin proteasome were enriched amongst putative direct targets of DVE-1. Follow-up studies indicated at least three different putative E3 ubiquitin ligases identified from the ChIP-seq analysis have altered expression in *dve-1* mutants. Together, these studies suggest that DVE-1 impacts synapse elimination at least in part through the regulation of pathways for protein degradation [64].

**Figure 3:**
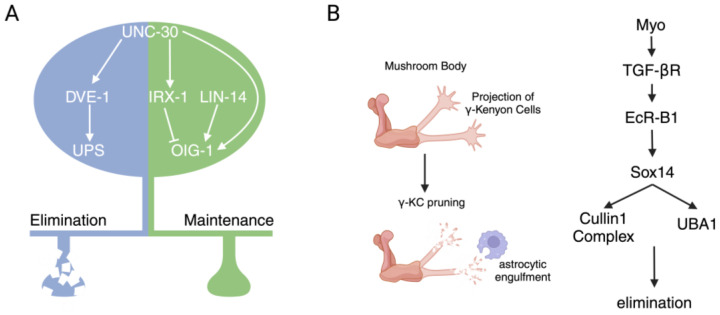
Transcriptional mechanisms controlling synapse elimination in *C. elegans* and *Drosophila.* (**A**) Left, transcriptional pathways regulating the elimination or stabilization of synaptic inputs to *C. elegans* DD GABAergic neurons. The Pitx transcription factor UNC-30 controls pathways for both synapse stabilization and elimination. The elimination of juvenile connections (blue shading) is dependent on the homeodomain transcriptional regulator DVE-1, likely through transcriptional control of ubiquitin–proteasome signaling (UPS). Synaptic stabilization (green shading) is regulated through temporally controlled transcription of the Ig domain family member *oig-1* by the Iroquois-like transcription factor IRX-1 and the BEN domain transcription factor LIN-14. *oig-1* expression is high in DD neurons prior to the onset of remodeling, leading to synapse stabilization. *oig-1* expression is downregulated in DD neurons with the onset of remodeling. (**B**) Transcriptional pathways regulating the pruning of γ-Kenyon cell (γ-KC) axons of the *Drosophila* mushroom body during metamorphosis. Left, schematic depicting the pruning of γ-KC axons and the clearance of axonal debris by astrocytes following pruning. Right, signaling pathway important for γ-KC pruning. γ-KC expression of the ecdysone receptor subunit EcR-B1 is upregulated through activation of TGF-β receptors by the astrocyte secreted ligand Myoglianin (Myo). Ecdysone activation of the ecdysone receptor complex results in the upregulation of the transcription factor Sox14 and several UPS components implicated in synapse elimination, including Cullin1 and UBA1.

*oig-1* encodes a protein containing a single immunoglobin(Ig)-like domain and is the only gene identified to date that is clearly implicated in both pre- and postsynaptic remodeling. *oig-1* mutants exhibit precocious remodeling, suggesting that OIG-1 normally acts to antagonize remodeling [42,44]. Specific expression of wild-type *oig-1* in the GABA neurons of *oig-1* mutants rescued precocious remodeling [42], indicating that *oig-1* acts cell-autonomously in DD neurons to impact the timing of remodeling. *oig-1* expression in DD neurons is tightly regulated through the combined actions of multiple transcriptional regulators including IRX-1, LIN-14, and UNC-30 [42,44] (Figure 3A). A decrease in *oig-1* expression is coincident with the onset of remodeling, suggesting that the downregulation of OIG-1 levels is an important step in the initiation of remodeling. While the precise mechanism of action of OIG-1 remains unclear, the Ig-like domain may enable the stabilization of presynaptic release sites and postsynaptic specializations through protein–protein interactions.

Following the reorganization of DD neuron synaptic contacts during remodeling, DD neurons undergo a final stage of maturation where finger-like protrusions from the ventral DD dendrites become evident [23,25,65]. These dendritic structures share many features with the dendritic spines found on mammalian neurons. Most notably, postsynaptic neurotransmitter receptor clusters are organized at their tips apposed to presynaptic release sites, and F-actin assemblies are localized to the spine neck. Intriguingly, a candidate screen to identify adhesion proteins required for receptor clustering on dendritic spines showed that spines are absent from mature animals that lack the synaptic organizer *nrx-1*/Neurexin [26]. Further analysis showed that the expression of *nrx-1* is required in presynaptic cholinergic neurons for the maintenance of spines on DD GABA dendrites. In the absence of *nrx-1*, dendritic spines emerge initially, but subsequently collapse in the absence of neurexin-mediated trans-synaptic adhesion [23,65].

An overall picture emerges from studies of the DD motor circuit, where the juvenile synaptic arrangement is stabilized at least partially through the regulated expression of the Ig domain protein OIG-1. Transcriptional regulation sets the timing of presynaptic remodeling by both downregulating *oig-1* expression and by mobilizing the expression of genes required for the removal of presynaptic release sites. Neuronal activity refines this timing and initiates specific steps in the removal program, such as bulk endocytosis of presynaptic material. Cytoskeletal regulation and protein trafficking are important for the relocation of presynaptic material to newly established synaptic release sites in the mature circuit. Though our understanding of postsynaptic remodeling in DD neurons is less well developed, many aspects appear distinct from those implicated in presynaptic remodeling; however, here too, OIG-1 plays a stabilizing role in the juvenile circuit. Transcriptional regulation through the homeodomain protein DVE-1 is important for postsynaptic removal. However, control of *dve-1* expression does not appear to be the central trigger for the process, raising the question of how postsynaptic removal is initiated. Moreover, how central events in pre- and postsynaptic rearrangements are coordinated at the cellular level remains an intriguing question.

### 3.4. Remodeling of C. elegans Synapses in Other Neuronal Classes and Contexts

While GABAergic DD neurons remain the most well-studied example in *C. elegans*, the synaptic connections of many other classes of *C. elegans* neurons are shaped through extensive remodeling. For example, mature synaptic connections between the HSN hermaphrodite-specific neuron and vulval muscles are sculpted through developmental synapse elimination. In this case, synapse elimination occurs through UPS-mediated protein degradation and involves a Skp1-cullin-F-box (SCF) E3 ubiquitin ligase, composed of SKR-1 and the F-box protein SEL-10 [66]. The positioning of HSN synapses is determined initially by interactions between the immunoglobulin superfamily (IgSF) protein SYG-1/NEPH1 and SYG-2, other IgSF protein expressed in epithelial guidepost cells [67,68]. Synapses that neighbor SYG-1/SYG-2 complexes are protected from elimination by SYG-1 binding of SKR-1 and inhibition of SCF complex assembly [66], suggesting that the specificity of synapse elimination is regulated subcellularly through spatially defined ubiquitin-mediated protein degradation.

Though synaptic remodeling has been studied most extensively in the context of *C. elegans* hermaphrodite neural development, it is important to note that synaptic remodeling also has critical roles in establishing sexually dimorphic wiring across *C. elegans* hermaphrodites and males. Prior to sexual maturation, many neurons have patterns of hybrid connections that are characteristic of both males and hermaphrodites. Sex-specific programs for synapse maintenance or pruning then produce sex-specific patterns of connectivity that are specified by the sexual identity of the pre- and postsynaptic partner neurons [69,70,71]. Conserved molecular factors, including the *C. elegans* netrin receptor UNC-40/DCC and the E3 ligase SEL-10/FBW7, have been implicated in sex-specific synaptic maintenance and pruning, respectively. For instance, SEL-10 activity is important in the elimination of hermaphrodite synapses between sensory neurons (PHB) and head interneurons (AVG) during sexually dimorphic rewiring. *sel-10* and *skr-1* mutant hermaphrodites each fail to eliminate their PHB-AVG synapses, while males are unaffected [70]. In contrast, males carrying a mutation in the netrin receptor *unc-40* undergo partial PHB-AVG synapse elimination while *unc-6*/Netrin mutant males eliminate PHB-AVG synapses completely [70]. Based on these and related findings, a model emerges where SEL-10-dependent elimination of PHB-AVG synapses occurs in hermaphrodites through the ubiquitination and degradation of UNC-40 and the loss of an UNC-40-dependent synaptic maintenance signal. Thus, sex-specific synaptic protein degradation helps to sculpt sexually dimorphic synaptic connectivity. Through genetic analysis of remodeling in DD neurons and other neuronal cell types, we have gained a new mechanistic understanding that informs studies of neurodevelopment across both invertebrate and vertebrate systems.

## 4. The Remodeling of Drosophila Neural Circuits Involves Neuron-Intrinsic and -Extrinsic Mechanisms

In contrast to the remodeling of *C. elegans* GABAergic motor neurons where primarily neuron-intrinsic events direct synapse removal and growth, many instances of circuit remodeling in *Drosophila* rely heavily on both intrinsic and extrinsic mechanisms. One of the most well-studied examples of *Drosophila* synaptic remodeling occurs in neurons of the mushroom body (MB). The circuits of the *Drosophila* MB have well-characterized roles in olfactory learning and memory and comprise three classes of neurons, known as Kenyon cells (KCs), which are born sequentially. The γ-KCs are the first-born and undergo stereotypic remodeling during metamorphosis, when γ-KC dendrites are eliminated and axons are pruned [11,72,73]. γ-KC axons and dendrites regrow in the pupal stage, starting roughly 18 h after puparium formation to form adult-specific connections [11,72,73].

### 4.1. Neuron-Intrinsic Mechanisms Direct Mushroom Body Remodeling

Similar to *C. elegans*, cytoskeletal regulation appears critical for the remodeling of fly γ-KCs. One of the earliest intrinsic events in the degenerative phase of γ-KC remodeling is the loss of MTs from axons. *α*-tubulin is widely distributed in the axons of γ-KCs during the late larval stages, but then is lost from the axonal segments that undergo eventual pruning [74], suggesting that the regulation of MT stability may be critical in defining axonal segments that will degenerate or be preserved.

The pruning of γ-KC axons requires the heterodimeric nuclear hormone receptor complex composed of Ultraspiricle and Ecr-B1 that is activated in response to the ecdysone hormone [75]. Pruning is limited to γ-KCs in part by the cell type-specific regulation of EcR-B1 expression where TGF-β signaling promotes the expression of EcR-B1 in γ-KCs, but not other MB cell types [76] (Figure 3B). The ubiquitin–proteasome system (UPS) has a central role in the pruning of γ-KC axons. The transcript levels of several UPS components, including the ubiquitin-activating enzyme Uba1, the SCF E3 ubiquitin ligase component Cullin1, and the Rpn6 subunit of the 19S proteasome regulatory particle are regulated through EcR-B1 signaling [77,78] and by the associated expression of the Sox14 transcription factor [78] (Figure 3B). However, the specific substrates that are degraded by the UPS to promote MB axon pruning remain unclear. Recent work has provided evidence that silencing of neuronal activity is also important for the pruning of γ-KC axons. Both external inhibitory input from presynaptic GABAergic APL neurons and γ-KC expression of the inward rectifying potassium channel 1, Irk1, were implicated in the inhibition of γ-KC activity, leading to pruning [79]. Interestingly, the destabilization of cell adhesion also appears critical for axonal pruning to proceed [80]. In particular, a reduction in membrane levels of the cell adhesion molecule Fasciclin II (FasII), an ortholog of the mammalian neural cell adhesion molecule (NCAM), through the Drosophila Jun Kinase Bsk was shown to be required for MB axon pruning [80]. Notably, cell adhesive mechanisms also have important roles in the re-growth of γ-KC axons following pruning. For example, expression of the Immunoglobulin superfamily protein Dpr12 is critical for axonal regrowth [81].

### 4.2. Extrinsic Factors Also Shape the Remodeling of Mushroom Body Gamma Neurons

Extrinsic mechanisms that impact the remodeling of γ-KCs are mediated in large part by the actions of glial cells. Glia secrete the Myoglianin ligand that binds to TGF-β receptors on γ-KCs to induce expression of EcR-B1 and trigger the axonal pruning program (Figure 3B). Glia also have a major role in the clearance of cellular debris following axon fragmentation. In particular, astrocytes have been shown to invade sites of degeneration and engulf cellular debris (Figure 3B) through a mechanism that employs the engulfment receptor Draper (CED-1/Drpr) and CED-6 for the clearance of axonal fragments [82] and subsequent lysosomal degradation [83,84].

## 5. Shared Features of Invertebrate and Vertebrate Neuronal Remodeling

Similar to *C. elegans* and *Drosophila*, vertebrate circuits in both the central (CNS) and peripheral nervous system (PNS) undergo extensive developmental remodeling. Remarkably, many of the mechanisms discussed here that are important for invertebrate circuit remodeling have parallels in the remodeling of mammalian and non-mammalian vertebrate circuits. These mechanistic parallels identify key conserved processes at the core of diverse remodeling programs (Table 2) and fall into the following broad categories: (1) transcriptional mechanisms for the cell-autonomous control of axon/synapse elimination and growth pathways; (2) protein degradation through the ubiquitin–proteasome system; (3) cytoskeletal reorganization; (4) regulation of intercellular adhesion; and (5) glial clearance of cellular debris.

For both *C. elegans* and *Drosophila*, circuit remodeling is regulated through cell-autonomous transcriptional control of the genes involved in synapse elimination and rewiring. Transcriptional regulation of the UPS has a particularly prominent role. For instance, ecdysone signaling regulates the expression of key UPS pathway genes important for axon pruning in *Drosophila*. Similarly, the elimination of juvenile postsynaptic structures in *C. elegans* DD GABAergic neurons requires transcriptional regulation by the homeodomain protein DVE-1 [20]. Components of the UPS pathway are enriched amongst the putative directs targets of DVE-1 and animals carrying a mutation in the sole *C. elegans* E1 ubiquitin ligase, *uba-1*, experience a significant delay in synapse elimination [20]. These processes are broadly paralleled by similar cell-autonomous mechanisms for transcriptional control of synapse elimination in mammalian neurons. For example, in vitro and in vivo studies implicate the activation of the myocyte enhancer factor 2 (MEF2) transcription factor in synapse elimination in mammalian hippocampal CA1 neurons [85,86]. MEF2 activation triggers the synaptic accumulation of the E3 ubiquitin ligase Mdm2, and subsequent Mdm2-dependent ubiquitination of the postsynaptic scaffold PSD-95. In parallel, MEF2 activation was shown to induce transcription of the protocadherin *Pcdh10*, which mediates synapse elimination in part by promoting the association of the ubiquitinated form of PSD-95 with the proteasome [87,88].

The regulation of MT dynamics in *C. elegans* remodeling and MT disassembly in *Drosophila* axon pruning highlight the importance of cytoskeletal remodeling in these systems. Cytoskeletal remodeling is also a key feature in axon pruning at the developing mammalian neuromuscular junction (NMJ). During neuromuscular synaptogenesis, multiple motor axon branches converge on the same postsynaptic muscle target [89]. Muscle innervation by a single motor axon branch is achieved through activity-dependent competition and the elimination of weaker connections [90,91,92]. Recent in vivo studies showed that the loss of microtubules is a major determinant of branch-specific axon loss during synapse elimination, in part mediated through the actions of the microtubule-severing protein spastin [93].

Cell-specific regulation of adhesive mechanisms is another common feature of remodeling across worms, flies, and mammals. Neuron-intrinsic regulation of adhesive mechanisms have been shown to be important for remodeling in worms and flies, while glial regulation of adhesion has been implicated in the refinement of the mammalian NMJ. For instance, temporally controlled expression of the *C. elegans* IgSF protein OIG-1 stabilizes juvenile synapses on DD neurons prior to remodeling and downregulation of OIG-1 stabilization is an important cue for the initiation of remodeling [42]. Similarly, reduced membrane expression of the Drosophila IgSF protein FasII is important for the progression of MB remodeling [44,80]. In mammals, the loss of glial Neurofascin significantly delays synapse elimination during synapse refinement at the NMJ. Interestingly, the effect on synapse elimination is mediated in part through glial adhesive regulation of cytoskeletal organization in the axons of motor neurons in vivo [94].

Glial clearance of neuronal debris is a shared feature of remodeling programs in both mammalian circuits and Drosophila MB. While the remodeling of *C. elegans* DD GABAergic neurons occurs without significant alterations in DD neuron structure and proceeds without glial involvement, glial phagocytosis has been implicated in the sculpting of *C. elegans* peripheral sensory endings [95]. Thus, glial engulfment of neuronal material is a shared feature across each of these systems. Astrocyte engulfment and elimination of synaptic material in the mouse retinogeniculate system shares particularly striking parallels with glial engulfment of axon fragments during *Drosophila* MB remodeling [82,96,97]. In each case, phagocytosis is mediated through activation of the highly conserved MEGF10/Draper protein, initially identified from studies of apoptotic cell clearance in *C. elegans* (CED-1) [82,96,97]. Similarly, in vivo studies have demonstrated that axon trogocytosis by microglia is important for pruning in the developing retinotectal system of the non-mammalian vertebrate *Xenopus laevis* [98].

**Table 2:** Examples of genes implicated in select processes important for synapse remodeling across systems.

Conserved Processes in Synapse Remodeling			
Process	*Caenorhabditis elegans*	*Drosophila melanogaster*	Mammalian
Transcription	LIN-14, IRX-1, DVE-1, HBL-1 [20,22,31,32,33,34,35,36,42,43,47]	Sox14 [78]	MEF2 [86,87,88]
Protein degradation	SEL-10 E3 ligase, UBA-1, others [20,66,70]	Uba1, Cullin1, Rpn6 [77,78]	Mdm2 E3 ligase [87,88]
Cytoskeletal reorganization	UNC-104/Kinesin-3, CDK-5/Cdk5 [62]	Efa6, Stai [99]	Spastin [93]
Intercellular adhesion	OIG-1/single Ig-domain protein [42,44]	FasII/Fasciclin [80]	Neurofascin [94]
Engulfment	Not required for DD remodeling	Draper [83,84,85]	MEGF10 [100]

## 6. Future Prospects

The relative ease of applying traditional forward genetic screening approaches in invertebrate systems remains a powerful asset for identifying new genetic pathways important for synaptic remodeling. However, a number of new technical innovations have enhanced our ability to investigate the molecular mechanisms that drive neural circuit remodeling and their associated impacts on synaptic connectivity. Recent advances in the development of optical tools for improved visualization of cell morphological features and for spatiotemporally controlled genetic manipulation of both neurons and glia have dramatically enhanced our ability to detect synaptic alterations and to perturb the molecular pathways that give rise to them. The recent development of techniques for stable labeling of presynaptic sites in the non-mammalian vertebrate model, *Danio rerio*, offers promise for in vivo studies of developmental synaptic remodeling that will complement those in both invertebrate and mammalian vertebrate models [101]. The emergence of techniques for CRISPR/Cas9-mediated genome editing [102,103,104,105] has enabled the widespread use of refined genetic tools, such as split GFP, to provide endogenous cell-specific labelling of neurons and synapses [106,107]. Continuing improvements in technologies for both super-resolution and light sheet microscopy, as well as live imaging, have accelerated the pace of discovery and will continue to fuel further advances in the field [108,109,110,111]. New technologies for cell- and tissue-specific transcriptomics have dramatically improved our ability to detect molecular changes underlying neuronal remodeling and to understand their temporal regulation. Recent efforts from the *C. elegans* Neuronal Gene Expression Map & Network (CeNGEN) consortium have delivered a nervous system-wide atlas of gene expression through the use of single cell RNA-sequencing (scRNA-seq) [112,113,114], advancing the potential for defining the transcriptional landscape of individual neuron types at precisely defined periods of neurodevelopment. New methodologies for spatiotemporally controlled genetic perturbation, such as the auxin inducible degron (AID) [20,115,116] and flippase (FLP)–FRT recombinant systems [117,118], offer enhanced resolution for future efforts to elucidate the molecular underpinnings of neural circuit connections and remodeling. These molecular approaches are complemented by collaborative efforts to generate nervous system-wide atlases of expression and interactions [119,120,121]. The strengths of invertebrate models outlined in this review, in conjunction with the continued development of new technologies, provide great promise toward an increasingly comprehensive understanding of this critically important neurodevelopmental process.

## Figures and Tables

**Table 1:** Overview of genes implicated in DD synapse remodeling.

Gene Name	Function in DD Remodeling	Reference
*lin-14*	Transcription factor (delays presynaptic remodeling)	[31,32,33,34,35,47]
*hbl-1*	Transcription factor (promotes presynaptic remodeling)	[36]
MYRF family genes	Transcription factors (promote remodeling)	[45,46,47]
*unc-55*	Transcription factor (suppress remodeling)	[36,40,41,42,43,48,49]
*unc-30*	Transcription factor (GABAergic neuronal identity) Timing of remodeling and synapse patterning	[37,38,39,40,41,42,43,44]
*irx-1*	Transcription factor (promotes DD remodeling)	[22,42,43]
*unc-8*	ENaC channel (removal of presynaptic sites from DDs)	[22,43,50,51,52]
*oig-1*	Ig domain protein (antagonizes pre- and postsynaptic remodeling)	[42,44]
*dve-1*	Homeodomain protein (elimination of juvenile DD synaptic inputs)	[20]
